# Early oral protein-containing diets following elective lower gastrointestinal tract surgery in adults: a meta-analysis of randomized clinical trials

**DOI:** 10.1186/s13741-021-00179-3

**Published:** 2021-03-23

**Authors:** Hong Pu, Philippa T. Heighes, Fiona Simpson, Yaoli Wang, Zeping Liang, Paul Wischmeyer, Thomas J. Hugh, Gordon S. Doig

**Affiliations:** 1 Northern Clinical School Intensive Care Research Unit, Faculty of Medicine and Health, University of Sydney, Kolling Building—RNSH, Pacific Hwy, St Leonards, NSW 2065 Australia; 2 Department of Critical Care Medicine, West China Hospital of Sichuan University, Chengdu, People’s Republic of China; 3 Nutrition Services, Royal North Shore Hospital, Sydney, Australia; 4 Department of Critical Care Medicine, Daping Hospital, Chongqing, People’s Republic of China; 5 Department of Anesthesiology and Surgery, Duke University, Durham, NC USA; 6 Upper GI Surgical Department, Royal North Shore Hospital and the University of Sydney, Sydney, Australia

**Keywords:** Postoperative nutrition, Protein, Elective surgery, Meta-analysis, Mortality

## Abstract

**Background:**

Although current guidelines make consensus recommendations for the early resumption of oral intake after surgery, a recent comprehensive meta-analysis failed to identify any patient-centered benefits. We hypothesized this finding was attributable to pooling studies providing effective protein-containing diets with ineffective non-protein liquid diets. Therefore, the aim of this paper was to investigate the safety and efficacy of *early oral protein*-*containing diets* versus later (traditional) feeding after elective lower gastrointestinal tract surgery in adults.

**Methods:**

PubMed, Embase, and the China National Knowledge Infrastructure databases were searched from inception until 1 August 2019. Reference lists of retrieved studies were hand searched to identify randomized clinical trials reporting mortality. No language restrictions were applied. Study selection, risk of bias appraisal and data abstraction were undertaken independently by two authors. Disagreements were settled by obtaining an opinion of a third author. Majority decisions prevailed. After assessment of underlying assumptions, a fixed-effects method was used for analysis. The primary outcome was mortality. Secondary outcomes included surgical site infections, postoperative nausea and vomiting, serious postoperative complications and other key measures of safety and efficacy.

**Results:**

Eight randomized clinical trials recruiting 657 patients were included. Compared with later (traditional) feeding, commencing an early oral protein-containing diet resulted in a statistically significant reduction in mortality (odds ratio [OR] 0.31, *P* = 0.02, *I*^2^ = 0%). An early oral protein-containing diet also significantly reduced surgical site infections (OR 0.39, *P* = 0.002, *I*^2^ = 32%), postoperative nausea and vomiting (OR 0.62, *P* = 0.04, *I*^2^ = 37%), serious postoperative complications (OR 0.60, *P* = 0.01, *I*^2^ = 25%), and significantly improved other major outcomes. No harms attributable to an early oral protein-containing diet were identified.

**Conclusions:**

The results of this systematic review can be used to upgrade current guideline statements to a *grade A recommendation* supporting an *oral protein*-*containing diet* commenced before the end of postoperative day 1 after elective lower gastrointestinal surgery in adults.

**Supplementary Information:**

The online version contains supplementary material available at 10.1186/s13741-021-00179-3.

## Background

Early oral intake after elective surgery is considered to be “safe and vital for optimizing postoperative outcomes” (Wischmeyer et al., 2018). Meta-analyses have shown that, compared to later (traditional) feeding, early oral intake after elective colorectal surgery *may* significantly reduce postoperative infections (Lewis et al., 2001), serious postoperative complications (Osland et al., 2011), anastomotic leaks (Smeets et al., 2018), and mortality (Lewis et al., 2009). However, the most comprehensive systematic review and meta-analysis conducted by leading experts on the topic, updated in 2019, did attribute a significant reduction in hospital stay to early oral intake but it failed to confirm any of these previously reported important patient-centered clinical benefits (Herbert et al., 2019). All of these previous meta-analyses based their conclusions on a pooled assessment of *protein*-*containing* diets with *non*-*protein* liquid diets.

Early initiation of a protein-containing diet has been shown to significantly reduce mortality after urgent or emergency surgery for major trauma (Doig et al., 2011) and major burn injury (Pu et al., 2018). Furthermore, pneumonia, sepsis, gastrointestinal hemorrhage, and duration of hospital stay are also significantly reduced in these surgical populations (Doig et al., 2011; Pu et al., 2018). We were unable to find any meta-analyses that explicitly focused on the benefits of an early oral protein-containing diet after elective surgery.

The purpose of this systematic review was to identify, appraise, and synthesize evidence from randomized clinical trials (RCTs) evaluating the impact of an early oral protein-containing diet, compared to later (traditional feeding), on outcomes after elective lower gastrointestinal tract surgery in adults. The primary outcome for meta-analysis was mortality.

## Materials and methods

This study was conducted and reported in compliance with established methodological guidelines (Moher et al., 2009). Detailed study methods were published online in advance of search close-out (Pu et al., 2019).

Study selection, risk of bias appraisal, and data abstraction were undertaken by two authors. Disagreements were settled by obtaining an opinion of a third independent author. Majority decisions prevailed.

### Literature search

MEDLINE (www.PubMed.org), Embase (www.EMBASE.com), and the China National Knowledge Infrastructure (www.CNKI.com.cn) were searched from inception until 1 August 2019. Appropriate database specific statements and terms (Doig et al., 2011; Pu et al., 2018) are reported in the Online-only Supplement. Reference lists of retrieved papers were hand searched.

### Study selection

All RCTs comparing early oral or enteral nutrition to later (traditional) feeding published in any language were retrieved in full and screened for inclusion. The intervention of interest was defined as oral or enteral intake initiated within 24 h of surgery using a drink, food, or solution that contained calories *and* protein. The comparison group was accepted to include any form of nutrition commenced later than 24 h after surgery. When needed, end of postoperative day [POD] 1 was used to define the outer limit for this 24 h period.

RCTs reporting mortality conducted in adult patients who had received surgery to the lower gastrointestinal tract (distal to the ligament of Treitz) were eligible for inclusion and were reviewed in detail.

### Risk of bias

Included trials were appraised on the reporting of three key methodological criteria: (1) maintenance of allocation concealment, (2) use of blinding, and (3) completeness of follow-up. Major flaws leading to a recognized high risk of bias were defined a priori as clear failure to maintain allocation concealment (Higgins, 2011) and excessive (> 10%) loss to follow-up (Graf et al., 2002)

### Outcomes

The primary outcome was mortality, assessed at the longest reported follow-up interval. Secondary outcomes included physical function, quality of life, duration of hospital stay, requirement for intensive care unit (ICU) admission, surgical site infections, anastomotic leak/dehiscence, postoperative nausea and vomiting, pneumonia, and need for re-operation. Number of patients with intra-abdominal abscess/peritonitis, severe postoperative complications, and postoperative infections were also assessed.

### Statistical analysis

The Mantel-Haenszel method was used to calculate the odds ratio (OR) metric unless data was sparse, in which case the Peto method was used (Higgins, 2011; Bradburn et al., 2007). The underlying assumption behind the fixed-effects model was assessed with a formal chi-square test of heterogeneity (Villar et al., 2001) and quantified using the *I*^2^ metric (Higgins & Thompson, 2002). Important heterogeneity was defined as a *P* value for the test of heterogeneity (*P*_*heterogeneity*_) less than 0.10 or *I*^2^ greater than 50% (Hatala et al., 2005). Publication bias was assessed using a Funnel plot of the primary outcome.

Analysis was conducted using RevMan Version 5.3.5 for Windows (The Cochrane Collaboration®, Oxford, England, 2014). A two-tailed *P* value less than 0.05 was accepted to indicate statistical significance.

### Sensitivity analysis

Focused on the primary outcome, the sensitivity analysis considered trials with *less certainty* regarding protein content of the intervention group’s early nutrition.

### Heterogeneity and stratified analysis

If important heterogeneity was detected, the following a priori identified potential sources of heterogeneity were investigated via stratified analysis: (1) methodological quality, (2) intervention timing and dose, (3) co-interventions and comparison intervention received, and (4) measurement and timing of outcomes (Glasziou & Sanders, 2002).

## Results

### Literature search and study selection

**Fig. 1:**
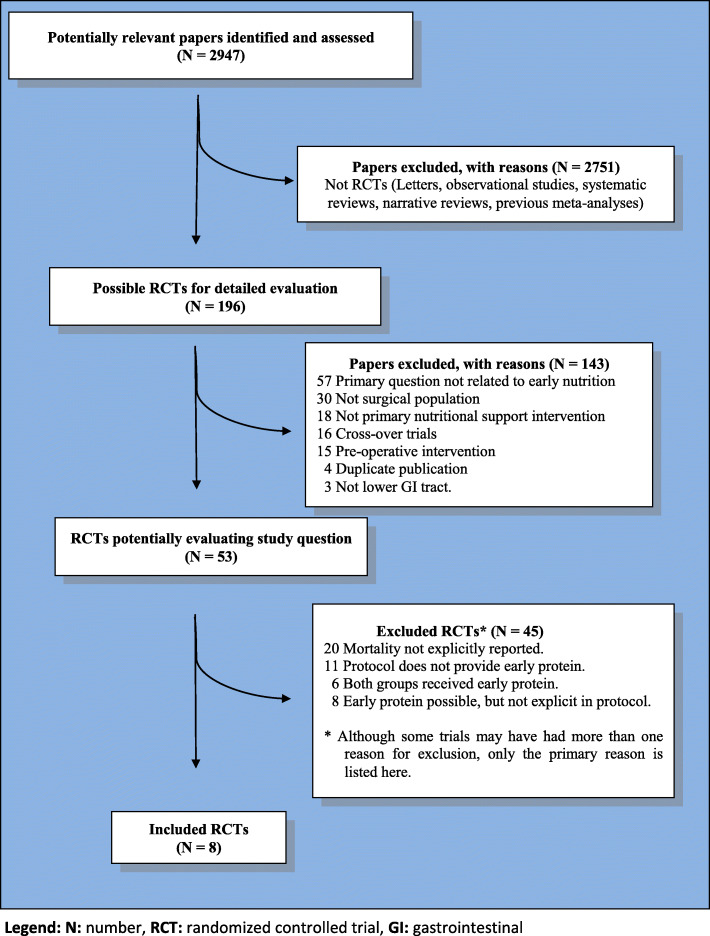
Flow diagram of the study selection process. N, number; RCT, randomized controlled trial; GI, gastrointestinal

The primary literature search identified 2947 abstracts of potentially eligible studies. Review of retrieved abstracts and hand searching of reference lists of published guidelines and systematic reviews resulted in 196 articles identified for retrieval. Of these 196 articles, 53 RCTs appeared to address key aspects of the primary study question. Eight of these 53 RCTs were deemed eligible for inclusion. Figure 1 reports the study selection flow. The Online-only Supplement provides additional details regarding RCTs deemed not eligible (eTable 1).

The eight included RCTs enrolled 657 participants (Beier-Holgersen & Boesby, 1996; Carr et al., 1996; Lau et al., 2014; Minig et al., 2009; Mulrooney et al., 2005; Ortiz et al., 1996; Shen, 2013; Stewart et al., 1998). Primary information regarding each of these eight RCTs was abstracted directly from the publications cited above. Additional information on three RCTs (Mulrooney et al., 2005; Ortiz et al., 1996; Stewart et al., 1998) were available from the systematic review by Herbert et al. (Herbert et al., 2019) Herbert et al. obtained these additional details by direct correspondence with the authors of these three RCTs.

Two included RCTs established early oral intake by using a protein drink (Beier-Holgersen & Boesby, 1996; Shen, 2013), two RCTs provided enteral nutrition via a feeding tube (Carr et al., 1996; Mulrooney et al., 2005), and four RCTs commenced a solid diet containing protein on POD 1 (Lau et al., 2014; Minig et al., 2009; Ortiz et al., 1996; Stewart et al., 1998). Details of the study populations and study interventions are reported in Table 1.

**Table 1:** Characteristics of included studies

Study	Patient population	Protocol for early nutrition intervention	Control
**Beier-Holgersen and Boesby,** 1996	60 patients with gastrointestinal disease undergoing abdominal surgery (87% underwent colorectal surgery).	**Start within 4 h of surgery (POD 0):** 60 mls of protein drink (Nutricia Nutridrink, Orange) every h, for total of 600 ml. **POD 1:** 1000 ml of protein drink; **POD 2:** 1400 ml protein drink; **POD 3 and 4:** 1800 ml protein drink.	Placebo (water with orange flavor) following same protocol as early protein drink group.
**Carr et al.,** 1996	28 patients undergoing intestinal resection distal to the ligament of Treitz.	**Start within 2 to 3 h of surgery (POD 0):** 25 ml/h EN (Fresubin, Fresenius) via N-J tube. Rate increased by 25 ml every 4 h until target of 35 ml/kg/day achieved.	NPO until passage of flatus.
**Lau et al.,** 2014	111 elective colorectal surgery patients.	**Start POD 1:** Solid diet that left minimal residue in the lower intestinal tract after digestion and absorption.	**Start POD 1:** Clear liquids, specifically omitting all solids (ex., milk, and fruit juice with pulp). **POD 2:** Advanced to solid diet.
**Minig et al.,** 2009	51 gynecologic oncology patients. (Over 70% also had rectosigmoid resection in addition to hysterectomy.)	**Start POD 0:** Clear fluids and other liquids (tea, apple juice, etc.). If fluids tolerated, progress to regular diet by end of POD 1.	NPO until resolution of ileus.
**Mulrooney et al.,** 2005	73 patients undergoing colonic resection	**Start within 24 h of surgery**: 25 ml/h EN (Nutrison Standard, Nutricia) via N-J tube; **POD 2:** 50 ml/h EN; **POD 3:** 75 ml/h: **POD 4:** EN increased to calculated nutritional targets.	NPO until passage of flatus.
**Ortiz et al.,** 1996	190 patients undergoing elective colon and rectal surgery.	Early removal of NG tube with **clear liquids on POD 0** followed by **regular diet on POD 1.**	NPO until resolution of ileus.
**Shen** 2013	82 patients with colorectal cancer undergoing surgery.	**Start POD 1:** 200 mls 0.9% Saline and 200 ml EN; **POD 2:** 500 to 1000 ml EN; **POD 3:** 1500 to 2000 ml of EN; **POD 4:** 2000 ml EN continued until POD 7.	**Start standard PN POD 2**: standard PN continued until POD 7.
**Stewart et al.,** 1998	88 patients undergoing elective colorectal resection with anastomosis, without stoma formation.	**Start within 4 h of surgery (POD 0):** free fluids; **POD 1:** Solid diet.	NPO until passage of flatus or bowel movement.

*POD*: postoperative day; *EN* enteral nutrition solution containing protein; *NPO* nil per os; *PN* parenteral nutrition solution containing glucose, amino acids, and protein; *N*-*J* naso-jejunal tube

### Risk of bias

Based on a priori defined criteria (Higgins, 2011; Graf et al., 2002), only one study was found to have a major methodological flaw resulting in a high risk of bias (Minig et al., 2009).

Three RCTs explicitly reported the process used to maintain allocation concealment (Carr et al., 1996; Lau et al., 2014; Minig et al., 2009) whilst the remaining five were unclear. One study achieved blinding using a placebo intervention (Beier-Holgersen & Boesby, 1996). Three studies documented failure to follow-up all randomized patients, with one reporting loss of 6.3% (7/111) (Lau et al., 2014) of randomized patients, a second reporting 9.1% (8/88) loss (Stewart et al., 1998), and the third documenting loss of 21.5% (11/51) of randomized patients (Minig et al., 2009). Complete results of the risk of bias assessment are presented in eFigure 1.

The funnel plot of the primary outcome did not reveal publication bias (eFigure 2).

### Mortality

Eight RCTs enrolling 657 patients were included in the analysis of mortality. Two studies reported mortality at study day 30 (Beier-Holgersen & Boesby, 1996; Lau et al., 2014), and one reported mortality at study day 60 (Mulrooney et al., 2005), with the remaining five studies reporting mortality at time of hospital discharge. Mortality data for the trial by Mulrooney et al. (Mulrooney et al., 2005) was abstracted from the systematic review by Herbert et al. (Herbert et al., 2019). Herbert et al. reported corresponding with Mulrooney et al. to obtain this mortality information.

**Fig. 2:**
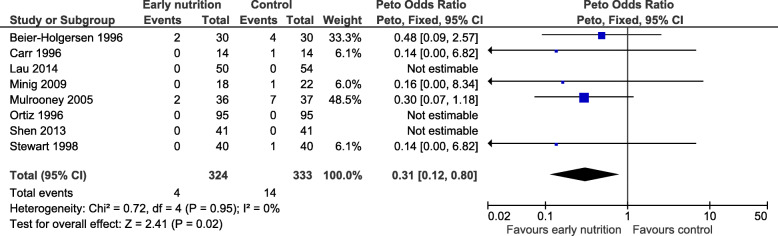
Analysis of primary outcome, mortality. CI, confidence interval

Compared with later (traditional) feeding, commencing an early oral protein-containing diet resulted in a statistically significant reduction in mortality (OR 0.31, 95% confidence interval [CI] 0.12 to 0.80, *P* = 0.02, Fig. 2), with no important heterogeneity detected (*P*_*heterogeneity*_ = 0.95, *I*^2^ = 0%).

### Physical function

Three studies reported measures of physical function. Carr et al. reported change in handgrip strength (Carr et al., 1996), Stewart et al. documented time to mobilization after surgery (Stewart et al., 1998), and Minig et al. assessed physical function at study day 30 using the European Organization for Research and Treatment of Cancer (EORTC) C-30 (Minig et al., 2009).

Carr et al. reported a mean 9.6 (standard deviation [SD] 2.1) kg loss in handgrip strength in control patients, and a 6.7 (3.2) kg loss in handgrip strength in patients who received an early diet containing protein (Carr et al., 1996). Assessed using a standard t-test for differences between groups, patients who received an early diet containing protein experienced significantly less handgrip strength loss (2.9 kg less, 95% CI 0.9 to 4.9 kg, *P =* 0.01).

Minig et al. failed to find a significant difference between groups with regards to physical function assessed at study day 30 using EORTC C-30 (76.1 ± 14.3 early vs. 62.1 ± 25.3 traditional, *P =* 0.146) (Minig et al., 2009).

Stewart et al. also failed to find a significant difference between groups with regards to time to mobilization after surgery (Stewart et al., 1998). Stewart et al. did not report the actual times to mobilization for each group.

Because of the differences in outcome metrics reported, these measures of physical function could not be pooled.

### Quality of life

One RCT reported formal measures of quality of life (Minig et al., 2009). Minig et al. failed to find a significant difference in EORTC OV-28 assessed at study day 30.

### Duration of hospital stay

All eight included RCTs reported duration of hospital stay. One trial reported differences in median hospital stay (Beier-Holgersen & Boesby, 1996), with the remaining seven reporting mean (SD). Mean (SD) hospital stay data for the trials by Minig et al., Mulrooney et al., and Stewart et al. was abstracted from the systematic review by Herbert et al. (Herbert et al., 2019) Herbert et al. reported corresponding with these authors to obtain this additional mean (SD) information regarding hospital stay.

Using a non-parametric test, the individual RCT conducted by Beier-Holgersen et al. reported a *trend* towards a reduction in median hospital stay for patients who received an early diet containing protein: median 8 vs. 11.5 days (*P* = 0.08) (Beier-Holgersen & Boesby, 1996).

Meta-analysis of the mean (SD) length of stay data reported by seven RCTs recruiting 598 patients demonstrated a significantly shorter hospital stay for patients randomized to receive an early oral protein-containing diet (− 2.12 days, 95% CI − 2.74 to − 1.49 days, *P <* 0.00001, eFigure 3); however, important heterogeneity was detected (*P*_*heterogeneity*_ = 0.00006, *I*^2^ = 75%). Sources of this heterogeneity are investigated further with stratified analysis (see later in the “Results” section).

### Intensive care unit admission

Three studies explicitly reported intensive care unit (ICU) admission rates after surgery (Beier-Holgersen & Boesby, 1996; Minig et al., 2009; Ortiz et al., 1996). Meta-analysis did not reveal any significant difference between groups (OR 0.61, 95% CI 0.24 to 1.53, *P* = 0.29, eFigure 4), with no important heterogeneity detected (*P*_*heterogeneity*_ = 0.55, *I*^2^ = 0%).

### Surgical site infections

All eight RCTs documented surgical site infection rates (Beier-Holgersen & Boesby, 1996; Carr et al., 1996; Lau et al., 2014; Minig et al., 2009; Mulrooney et al., 2005; Ortiz et al., 1996; Shen, 2013; Stewart et al., 1998); however, one combined reporting of surgical site infections with urinary tract infections and therefore could not be included in this pooled analysis (Carr et al., 1996). Surgical site infection data for the trial by Mulrooney et al. was abstracted from the systematic review by Herbert et al. (Herbert et al., 2019). Herbert et al. reported corresponding with Mulrooney et al. to obtain this additional information.

**Fig. 3:**
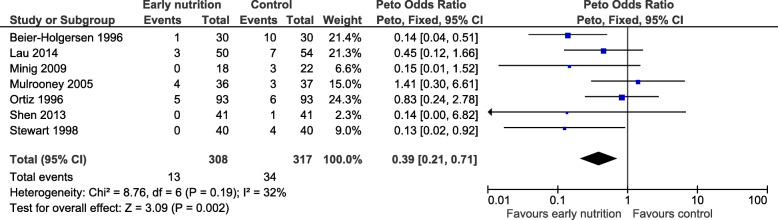
Number of patients with a surgical site infection. CI, confidence interval

The seven RCTs that explicitly reported surgical site infections enrolled 625 patients. Patients who received an early oral protein-containing diet were significantly less likely to experience a surgical site infection (OR 0.39, 95% CI 0.21 to 0.71, *P =* 0.002, Fig. 3), with no important heterogeneity detected (*P*_*heterogeneity*_ = 0.19, *I*^2^ = 32%).

### Anastomotic leak/dehiscence

Five RCTs enrolling 439 patients reported anastomotic leak (Beier-Holgersen & Boesby, 1996; Minig et al., 2009), anastomotic leak/dehiscence (Mulrooney et al., 2005; Stewart et al., 1998), or anastomotic breakdown (Ortiz et al., 1996). None of these trials reported using explicit and objective criteria to diagnose a leak/dehiscence/breakdown. Anastomotic leak/dehiscence data for the trial by Mulrooney et al. was abstracted from the systematic review by Herbert et al. (Herbert et al., 2019) Herbert et al. reported corresponding with Mulrooney et al. to obtain this additional information.

Meta-analysis did not find any significant difference between groups (OR 0.74, 95% CI 0.30 to 1.87, *P =* 0.53, eFigure 5) and no important heterogeneity was detected (*P*_*heterogeneity*_ = 0.10, *I*^2^ = 49%).

### Postoperative nausea and vomiting

Three studies reported nausea (Beier-Holgersen & Boesby, 1996; Lau et al., 2014; Stewart et al., 1998). Using a Likert scale, Lau et al. reported a significantly lower nausea score in the early diet containing protein group compared to the traditional group (2.5 early vs. 4.7, *P =* 0.01) (Lau et al., 2014). However, Stewart et al. failed to find a significant difference between groups using a visual-analog nausea scale (29 early vs. 31, reported as not significant [NS]) (Stewart et al., 1998), and Beier-Holgersen et al. also failed to find a significant difference between groups in the incidence of nausea (19/30 early vs. 22/30, NS) (Beier-Holgersen & Boesby, 1996). Due to the different measures used to assess nausea, pooled analysis could not be undertaken.

Three RCTs reported the number of patients who vomited (Beier-Holgersen & Boesby, 1996; Lau et al., 2014; Stewart et al., 1998), two RCTs reported the number of patients with “nausea and vomiting” combined (Carr et al., 1996; Minig et al., 2009), and one study provided a graphical representation of vomiting on each study day but did not explicitly report rates (Ortiz et al., 1996).

Pooled analysis of the five RCTs enrolling 312 patients that explicitly reported rates demonstrated a significant reduction in postoperative nausea and vomiting attributable to the provision of an early oral protein-containing diet (OR 0.62, 95% CI 0.38 to 0.99, *P =* 0.04, eFigure 6), with no important heterogeneity detected (*P*_*heterogeneity*_ = 0.17, *I*^2^ = 37%).

### Pneumonia

Six RCTs enrolling 585 patients reported pneumonia (Beier-Holgersen & Boesby, 1996; Lau et al., 2014; Mulrooney et al., 2005; Ortiz et al., 1996; Shen, 2013; Stewart et al., 1998). Pneumonia data for the trials by Mulrooney et al. and Stewart et al. was abstracted from the systematic review by Herbert et al. (Herbert et al., 2019). Herbert et al. reported corresponding with Mulrooney et al. and Stewart et al. to obtain this additional information.

Pooled analysis failed to demonstrate a significant difference between groups (OR 0.73, 95% CI 0.32 to 1.66, *P =* 0.45, eFigure 7), with no important heterogeneity detected (*P*_*heterogeneity*_ = 0.60, *I*^2^ = 0%).

### Need for re-operation

Three RCTs enrolling 204 patients explicitly reported need for re-operation (Beier-Holgersen & Boesby, 1996; Lau et al., 2014; Minig et al., 2009). Pooled analysis failed to demonstrate a significant difference between groups (OR 0.49, 95% CI 0.16 to 1.51, *P =* 0.22, eFigure 8), with no important heterogeneity detected (*P*_*heterogeneity*_ = 0.84, *I*^2^ = 0%).

### Intra-abdominal abscess/peritonitis

Six RCTs reported intra-abdominal abscess/peritonitis (Beier-Holgersen & Boesby, 1996; Lau et al., 2014; Minig et al., 2009; Mulrooney et al., 2005; Ortiz et al., 1996; Shen, 2013). Data for the trial by Mulrooney et al. was abstracted from the systematic review by Herbert et al. (Herbert et al., 2019). Herbert et al. reported corresponding with Mulrooney et al. to obtain this additional information.

Pooled analysis of these six RCTs enrolling 545 patients demonstrated a significant reduction in the onset of intra-abdominal abscess/peritonitis in patients who received an early oral protein-containing diet (OR 0.20, 95% CI 0.06 to 0.66, *P =* 0.008, eFigure 9), with no important heterogeneity detected (*P*_*heterogeneity*_ = 0.98, *I*^2^ = 0%).

### Number of patients with serious postoperative complications

Six RCTs enrolling 552 patients explicitly reported the total number of patients in each study group who had at least one serious postoperative complication (Beier-Holgersen & Boesby, 1996; Lau et al., 2014; Minig et al., 2009; Mulrooney et al., 2005; Ortiz et al., 1996; Shen, 2013; Stewart et al., 1998). Serious postoperative complications included acute myocardial infarction, anastomotic leak/dehiscence, unexpected return to surgery, hospital readmission within 30 days of discharge, surgical site infection, peritonitis, intestinal obstruction, and other postoperative infections. Because mortality served as the primary outcome for this study, it was not included in this analysis of serious postoperative complications.

Pooled analysis of these six RCTs revealed that the provision of an early oral protein-containing diet resulted in significantly fewer patients developing a serious postoperative complication (OR 0.60, 95% CI 0.40 to 0.89, *P* = 0.01, eFigure 10). Despite reporting of a different subset of serious postoperative complications by each study, there was no important heterogeneity detected (*P*_*heterogeneity*_ = 0.25, *I*^2^ = 25%).

### Number of patients with a postoperative infection

Four RCTs enrolling 210 patients reported the number of patients who had at least one postoperative infection (Beier-Holgersen & Boesby, 1996; Carr et al., 1996; Minig et al., 2009; Shen, 2013). Based on pooled analysis of these four RCTs, the provision of an early oral protein-containing diet resulted in a significant reduction in the number of patients who experienced a postoperative infection (OR 0.17, 95% CI 0.08 to 0.37, *P <* 0.0001, eFigure 11), with no important heterogeneity detected (*P*_*heterogeneity*_ = 0.83, *I*^2^ = 0%).

### Sensitivity analysis

Focused on the primary outcome, the sensitivity analysis considered trials with *less certainty* regarding protein content of the intervention group’s early nutrition. Thirteen clinical trials enrolling 1216 patients were identified for inclusion in the sensitivity analysis.

These RCTs described their early nutrition intervention as “water” (Wang et al., 2013), “5% glucose” (Cao, 2009; Li et al., 2006), “clear liquid” (Reissman et al., 1995; Pragatheeswarane et al., 2014), “allowed to drink” (Feo et al., 2004), “oral liquids” (Chatterjee et al., 2012), an “oral liquid diet” (da Fonseca et al., 2011), a “fluid diet” (Dag et al., 2011), “fluids” (El et al., 2009), a “liquid diet” (Hartsell et al., 1997), a “semi-fluid diet” (Lee et al., 2011), or “filtrate liquids” (Nematihonar et al., 2018).

**Fig. 4:**
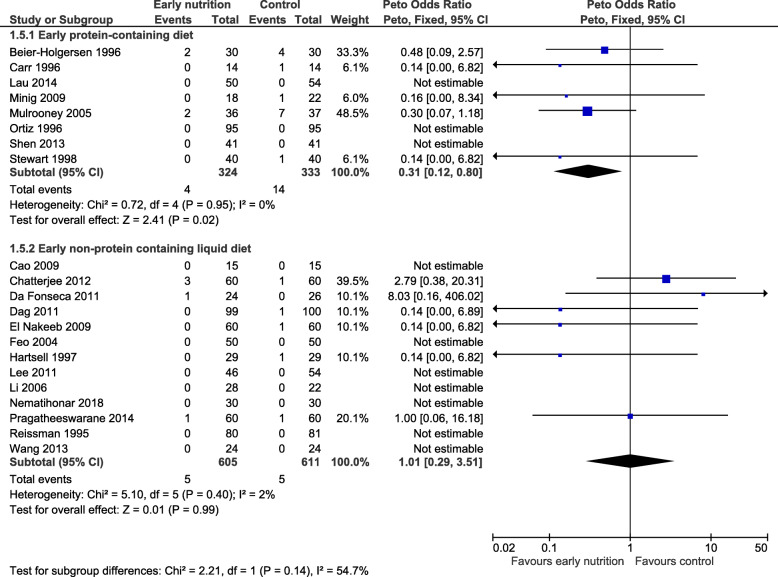
Sensitivity analysis: trials with less certainty regarding protein content. CI, confidence interval

Inclusion of these 13 RCTs in a sensitivity analysis failed to find an impact of early *non*-*protein liquid diets* on mortality (OR 1.01, 95% CI 0.29 to 3.51, *P =* 0.99). Furthermore, there was important heterogeneity *between* RCTs that evaluated a *protein*-*containing diet* and RCTs that evaluated *non*-*protein liquid diets*, suggesting these different interventions have different effects on mortality (*P*_*heterogeneity*_ = 0.14, *I*^2^ = 54.7%, Fig. 4).

### Heterogeneity and stratified analysis

The only statistically significant result demonstrating important heterogeneity was the analysis of duration of hospital stay (*I*^2^ = 75%, eFigure 3). Stratified analysis based on study intervention (enteral nutrition/solid diet/protein drink supplement) meaningfully reduced heterogeneity within each strata (eFigure 12). Interpretation of results within each strata revealed that early enteral nutrition did not have any effect on duration of hospital stay (1.05 days, 95% CI − 0.077 to 2.87 days, *P =* 0.26, *I*^2^ = 0%), whilst both an early solid protein-containing diet (− 1.86 days, 95% CI −2.73 to −1.00, *P <* 0.000001, *I*^2^ = 35%) and early use of protein drink supplements (− 3.54 days, 95% CI − 4.59 to − 2.49, *P <* 0.000001, *I*^2^ = 0%) significantly reduced duration of hospital stay.

## Discussion

This systematic review identified eight RCTs that evaluated the impact of an early oral protein-containing diet on outcomes after elective lower gastrointestinal tract surgery in adults. Compared to later (traditional) feeding, resumption of an oral protein-containing diet before the end of POD 1 significantly reduced mortality, surgical site infection rate, postoperative nausea and vomiting, serious postoperative complications, and other key measures of recovery after surgery. Furthermore, sensitivity analysis evaluating the early resumption of a *non*-*protein liquid diet* failed to find any effect on mortality.

### Mitochondrial biogenesis and recovery from abdominal surgery

Surgery to the lower gastrointestinal tract results in local tissue injury at the site of the external incision and also compromises gut barrier function, even with only mild intestinal handling (Alazawi et al., 2016; Anup et al., 1999). As a result of increased gut permeability, bacterial translocation occurs and initiates a systemic inflammatory response (Reddy et al., 2006; Qiao et al., 2009; Takesue et al., 2002; MacFie et al., 2006). Systemic inflammation arising from low-grade endotoxemia is known to impair mitochondrial function leading to a measurable reduction in energy production capacity (Vico et al., 2019). Mitochondrial dysfunction can depress protein synthesis (Morita et al., 2015) and impair immune response (West, 2017).

Mitochondrial biogenesis is the process of growth and replication undertaken by mitochondria in response to an increased need for energy production during metabolic stress (Holloszy, 2011). Serial muscle biopsies obtained from critically ill patients suggest that mitochondrial biogenesis may confer a survival advantage from critical illness by compensating for mitochondrial dysfunction and allowing increased energy demands to be met (Carre et al., 2010).

Amino acids are accepted to stimulate mitochondrial biogenesis by activating the mammalian target of rapamycin (mTOR) pathway (Morita et al., 2015; Dann & Thomas, 2006; Cao et al., 2019). The direct effect of early protein intake on the active up-regulation of mitochondrial biogenesis provides a plausible mechanism of action to explain the survival advantage, and other important outcome benefits, demonstrated in this meta-analysis (Carre et al., 2010).

### Anastomotic leak

Anastomotic leak is one of the most serious adverse events after colorectal surgery (Rahbari et al., 2010; Daams et al., 2014). Animal models demonstrate that initiating a protein-containing diet immediately after abdominal surgery significantly increases the *strength* of an ileal or colorectal anastomosis (Moss et al., 1980). Although our meta-analysis failed to find a significant reduction in the number of patients experiencing an anastomotic leak (eFigure 5), we did identify a significant reduction in the number of patients experiencing intra-abdominal abscess/peritonitis (eFigure 9). Based on the severity grading scheme proposed by Rahbari et al. (Rahbari et al., 2010), a reduction in intra-abdominal abscess/peritonitis suggests that an early oral diet containing protein may have reduced the *severity* (grade B or C) of anastomotic leaks. We strongly recommend that future trials in this field use pre-defined explicit criteria to objectively diagnose and grade the severity of anastomotic leaks.

### Duration of hospital stay

Due to the presence of substantial and important heterogeneity in the assessment of duration of hospital stay, pooled analysis of this outcome may be misleading (Glasziou & Sanders, 2002). Heterogeneity between trials can arise for a number of reasons, including real differences in the study intervention, patient populations and/or the severity/stage of disease studied by each trial. The purpose of stratified (grouped) analyses is to investigate these potential sources of important heterogeneity (Glasziou & Sanders, 2002).

When studies were stratified (grouped) based on intervention (enteral feeding tube vs. solid diet vs. protein drink supplement), we found that duration of hospital stay was significantly reduced by receiving an early solid diet or protein drink supplement, with no important heterogeneity detected within each strata (eFigure 12). However, since a number of stratified analyses were undertaken, these results should be viewed as hypothesis generating. We strongly recommend that future meta-analyses conduct assessments of duration of hospital stay stratified by intervention and interpret their results *within* each strata.

### The role of early nutrition in enhanced recovery after surgery programs

Early nutrition is considered to be an essential component of enhanced recovery after surgery (ERAS) programs (Ljungqvist et al., 2017). Striking recent data from a 911 patient observational study demonstrates that after undergoing colorectal cancer surgery in an ERAS program, delivery of nutrition on the day of operation was a strong independent predictor of 5-year postoperative survival (Gustafsson et al., 2016). This study does report that a significant number of patients received “nutritional supplements” on the day of surgery, but it does not record whether these supplements contained protein; thus, unfortunately, early protein intake was not completely assessed. Based on the results of our sensitivity analysis (Fig. 4), we found a mortality benefit of early protein intake, not early calories alone. Small observational studies have demonstrated that higher early protein intake by ERAS patients undergoing elective colorectal surgery is associated with a shorter duration of hospital stay (Yeung et al., 2017). Future studies in this field need to ensure that the effects of early protein intake are assessed more thoroughly.

### Which patients benefit the most?

It is intuitively appealing to attempt to identify individual patients who are most likely to benefit from an early oral protein-containing diet after elective lower gastrointestinal tract surgery. For example, if we could prove that patients who receive open operative procedures for malignant disease benefit most, we could focus our efforts on these patients. Unfortunately, the included clinical trials do not report outcomes by identifiable patient subgroups such as nutritional status, type of procedure, or specific underlying disease. However, because the analysis of our primary outcome (mortality) does not demonstrate any important heterogeneity (*P*_*heterogeneity*_ = 0.95, *I*^2^ = 0%), it supports the conclusion that there is no one subgroup of patients who benefit more than any other and that all patients included in this meta-analysis are likely to benefit in a similar fashion. Likewise, even though each clinical trial enrolled a different patient population who received different surgical procedures for different inciting causes, a lack of important statistical heterogeneity supports the conclusion that the overall benefit from early oral protein diets are similar across all of these studies.

### Strengths and limitations

The literature search supporting this systematic review was extensive and was not restricted by language of publication (Herbert et al., 2019). Although eight RCTs recruiting 657 patients were included, each RCT was relatively small in size and mortality was a sparse and rare outcome. Three of the included RCTs reported zero mortality events in each arm and one larger trial influenced 48.5% of the results. However, the Peto analytic method is accepted to provide unbiased results when outcomes are sparse (Higgins, 2011; Bradburn et al., 2007). Furthermore, significant benefits were demonstrated across multiple meaningful outcomes with no analysis suggesting any harm and other meta-analyses report a similar mortality benefit attributable to early postoperative initiation of a protein-containing diet in different surgical patient populations (Doig et al., 2011; Pu et al., 2018).

Whilst only one included RCT was identified as having a major methodological flaw leading to a potential high risk of bias (Stewart et al., 1998), all clinical trials in this field would benefit from improved reporting. Authors of RCTs should always report sufficient details regarding randomization such that the reader is assured that allocation concealment was maintained. Furthermore, outcomes should be reported on all patients randomized into an RCT and, finally, although blinding of a study intervention is not always feasible, blinded outcome assessment, and adjudication is always possible. The most recent CONSORT Statement serves as an excellent guide with regards to the minimum standards for reporting an RCT (Moher et al., 2012).

We did not assess time to first flatus, duration of ileus or time to first stool movement as we are unaware of any studies validating their use as surrogate measures for clinically meaningful outcomes (Prentice, 1989). Furthermore, we are unaware of any evidence-based statements from authoritative bodies or societies that make recommendations for their continued use to guide decisions to withhold nutrition from surgical patients (Wischmeyer et al., 2018).

Finally, it is important to note that the clinical trials included in this meta-analysis did not set out to determine the amount of protein that should be targeted over the first few postoperative days to achieve maximum benefit. Each of the included trials was pragmatic and attempted to achieve *any* protein intake during the first 24 h post-op. As in the real world, patients may refuse oral intake during the first 24 h. Due to the pragmatic nature of these trials, patients who refused oral intake were still included in the intention to treat analysis. Thus, the benefits established by these clinical trials are very likely to be achieved in the real world. Additional research is required to determine whether higher targets may benefit patients more.

## Conclusions

This first systematic review focused exclusively on the effects of an early oral protein-containing diet versus later (traditional) feeding following elective lower gastrointestinal tract surgery found eight RCTs that addressed this clinical question. Meta-analysis of these eight RCTs demonstrated a significant reduction in mortality and improvements in other key important outcomes, arising from an early oral protein-containing diet, with no indications of any harms. Furthermore, none of our analyses found any benefits in favor of later (traditional) feeding. We suggest our results support a *grade A recommendation* for an oral protein-containing diet to be initiated before the end of POD 1 after lower gastrointestinal surgery. If it is felt a *clear liquid* is clinically indicated, it is important to understand that *protein*-*containing clear liquid supplements are widely available*.

## Supplementary Information

**Additional file 1: **PubMed, Embase and CNKI search terms. **eTable 1.** RCTs excluded after detailed review. **eFigure 1.** Risk of bias summary figure. **eFigure 2.** Funnel plot for publication bias. Primary outcome (mortality). **eFigure 3.** Duration of hospital stay. **eFigure 4.** Need for ICU admission. **eFigure 5.** Anastomotic leak/dehiscence. **eFigure 6.** Postoperative nausea and vomiting. **eFigure 7.** Pneumonia. **eFigure 8.** Need for re-operation. **eFigure 9.** Number of patients with intra-abdominal abscess/peritonitis. **eFigure 10.** Number of patients with serious post-operative complications. **eFigure 11.** Number of patients with a post-operative infection. **eFigure 12.** Stratified analysis of duration of hospital stay

## Data Availability

All data generated or analyzed during this study are included in this published article, its supplementary information files, and the primary randomized controlled trials cited for inclusion.

## Abbreviations

CNKI: China National Knowledge Infrastructure
EORTC: European Organization for Research and Treatment of Cancer
