# Supplementary material for: Early oral protein-containing diets following elective lower gastrointestinal tract surgery in adults: a meta-analysis of randomized clinical trials
Source: Perioper Med (Lond). 2021 Mar 23;10:10. doi: 10.1186/s13741-021-00179-3 (PMC7986268; doi:10.1186/s13741-021-00179-3)
Supplement: Supplementary file 1 — Additional file 1: PubMed, Embase and CNKI search terms. eTable 1. RCTs excluded after detailed review. eFigure 1. Risk of bias summary figure. eFigure 2. Funnel plot for publication bias. Primary outcome (mortality). eFigure 3. Duration of hospital stay. eFigure 4. Need for ICU admission. eFigure 5. Anastomotic leak/dehiscence. eFigure 6. Postoperative nausea and vomiting. eFigure 7. Pneumonia. eFigure 8. Need for re-operation. eFigure 9. Number of patients with intra-abdominal abscess/peritonitis. eFigure 10. Number of patients with serious post-operative complications. eFigure 11. Number of patients with a post-operative infection. eFigure 12. Stratified analysis of duration of hospital stay [file 13741_2021_179_MOESM1_ESM.pdf]

THE UNIVERSITY OF SYDNEY

Online-Only Supplement:

Early oral protein-containing diets following elective lower gastrointestinal tract surgery in adults: A meta-analysis of randomized controlled trials.

Hong PU[a,b] (蒲虹), Philippa T. Heighes[a] and Gordon S. Doig[a]

From the [a]Northern Clinical School Intensive Care Research Unit, Faculty of Medicine and Health, University of Sydney, Australia and [b]Department of Critical Care Medicine, West China Hospital of Sichuan University, Chengdu, Peoples Republic of China.

Corresponding Author:

Dr. Gordon S. Doig,
Northern Clinical School Intensive Care Research Unit,
Faculty of Medicine and Health,
Kolling Building – RNSH
Pacific Hwy,
St Leonards
Australia 2065
Gordon.Doig@EvidenceBased.net
www.EvidenceBased.net/Research

16 Dec 2020

©2020 Gordon S. Doig, University of Sydney.

© 2020 Gordon S Doig, University of Sydney. All rights reserved. This publication is protected by copyright. No part of it may be reproduced for commercial purposes or distributed electronically without prior written permission of the publisher. Reproduction for personal or educational use is acceptable.

Contents

Title Page 1

PubMed, Embase and CNKI search terms 4

- PubMed search terms: 4
- Embase search terms: 4
- China National Knowledge Infrastructure search terms: 5

eTable 1: RCTs excluded after detailed review 6

eFigure 1. Risk of bias summary figure 12

eFigure 2. Funnel plot for publication bias. Primary outcome (mortality) 13

eFigure 3: Duration of hospital stay 13

eFigure 4: Need for ICU admission 13

eFigure 5: Anastomotic leak/dehiscence 13

eFigure 6: Postoperative nausea and vomiting 14

eFigure 7: Pneumonia 14

eFigure 8: Need for re-operation 14

eFigure 9: Number of patients with intra-abdominal abscess/peritonitis 14

eFigure 10: Number of patients with serious post-operative complications 15

eFigure 11: Number of patients with a post-operative infection 15

eFigure 12: Stratified analysis of duration of hospital stay 16

Reference List 17

©2020 Gordon S. Doig, University of Sydney.

PubMed, Embase and CNKI search terms

PubMed search terms:

The MEDLINE subject heading terms used to identify the nutritional support literature included:

nutrition therapy [MeSH terms] or nutritional support [MeSH terms] or nutrition phenomena [MeSH terms] or nutrition processes [MeSH terms] or nutrition disorders [MeSH terms] or nutrition assessment [MeSH terms]

These nutrition related terms were crossed with MEDLINE terms to restrict the results to RCTs conducted in patients undergoing large bowel surgery:

("lower gastrointestinal tract"[MeSH Terms] OR ("lower"[All Fields] AND "gastrointestinal"[All Fields] AND "tract"[All Fields]) OR "lower gastrointestinal tract"[All Fields]) OR ("intestine, large"[MeSH Terms] OR ("intestine"[All Fields] AND "large"[All Fields]) OR "large intestine"[All Fields] OR ("large"[All Fields] AND "bowel"[All Fields]) OR "large bowel"[All Fields]) OR ("colectomy"[MeSH Terms] OR "colectomy"[All Fields]) OR (("colostomy"[MeSH Terms] OR "colostomy"[All Fields]) AND colorectal[All Fields]) OR ("colon"[MeSH Terms] OR "colon"[All Fields]) OR ("administration, rectal"[MeSH Terms] OR ("administration"[All Fields] AND "rectal"[All Fields]) OR "rectal administration"[All Fields] OR "rectal"[All Fields]) OR ("rectum"[MeSH Terms] OR "rectum"[All Fields])

AND

("surgery"[Subheading] OR "surgery"[All Fields] OR "surgical procedures, operative"[MeSH Terms] OR ("surgical"[All Fields] AND "procedures"[All Fields] AND "operative"[All Fields]) OR "operative surgical procedures"[All Fields] OR "surgery"[All Fields] OR "general surgery"[MeSH Terms] OR ("general"[All Fields] AND "surgery"[All Fields]) OR "general surgery"[All Fields]) OR ("surgical procedures, operative"[MeSH Terms] OR ("surgical"[All Fields] AND "procedures"[All Fields] AND "operative"[All Fields]) OR "operative surgical procedures"[All Fields] OR "surgical"[All Fields]) OR operat$[All Fields] OR postoperati$[all]

AND broad MeSH terms to identify RCTs:

((clinical[Title/Abstract] AND trial[Title/Abstract]) OR clinical trials as topic[MeSH Terms] OR clinical trial[Publication Type] OR random*[Title/Abstract] OR random allocation[MeSH Terms] OR therapeutic use[MeSH Subheading])

Embase search terms:

The EMTREE terms used to identify the nutritional support literature included:

diet therapy/ or nutritional support/ or nutritional disorder/ or nutritional assessment/ or nutritional deficiency/ or feeding behaviour/ or nutritional status/ or overnutrition/

These nutrition related terms were crossed with EMTREE terms to restrict the results to RCTs conducted in large bowel surgery patients:

©2020 Gordon S. Doig, University of Sydney.

(random or clinical trial or randomised or randomized or randomized controlled trial or randomised controlled trial).mp

AND

(surgery or surgical or operat$ or post-operat$ or postoperat$).tw

AND

(lower gastrointestinal or large bowel or colectomy or colostomy or colorectal or colon or rectal or rectum).tw

China National Knowledge Infrastructure search terms:

The CNKI subject heading terms used to identify the nutritional support literature included:

营养生理学现象 or 营养疗法 or 营养支持 or 营养状况 or 营养评价 or 肠道营养 or 营养需要or 胃肠外营养 or 胃肠外营养，全 or 营养价值

AND

结肠切除术 or 直肠结肠切除术，重建性 or 结肠造口术

AND

随机分配 or 随机对照试验or 临床试验

©2020 Gordon S. Doig, University of Sydney.

eTable 1: RCTs excluded after detailed review.

Study	Patient population	Protocol specified early nutrition intervention	Reasons for exclusion
Boelens[1] 2014	Major rectal surgery	POD 1: on the day of surgery (day 0), 8 hours after ending the surgical procedure, **enteral nutrition (Nutrison Protein Plus Multi Fibre, Nutricia)** was started at 500 mL/24 hours. The next day (day 1 postoperative), at 8 o'clock in the morning, the continuous infusion was increased to 1 L/24 hours; on day 2, the infusion was increased to 2 L/24 hours. **Early EN compared to early PN (Kabiven Central with Dipeptiven, Fresenius Kabi)**. PN protocol same as EN protocol above.	Mortality not explicitly reported. Both groups received early protein.
Behrns [2] 2000	Elective intestinal surgery.	**Clear liquid diet** commenced on POD 2.	No early protein.
Binderow [3] 1994	Elective laparotomy with either a colonic or ileal resection.	**Regular solid food** on morning of POD 1.	Mortality not explicitly reported.
Cao [4] 2009	Postoperative colorectal cancer.	POD 1: **Began 5% Glucose** 500ml; POD 2: Enteral nutrition (Peptison, NUTRICIA) 500ml and 5% Glucose injection 500ml; POD 3: Peptison 500ml to 1500ml on the third POD, progressed to Peptison 100ml/h on the fourth to seventh POD.	No early protein.
Chatterjee [5] 2012	Gastrointestinal anastomosis and uncomplicated simple biliary-enteric anastomosis (choledochoduodenostomy) on an emergency or elective basis.	"**Oral liquids**" (25ml/hr) were started within 24 hours of operation in group A with clamping the NGT.	Early protein *possible,* but not explicit in protocol. 'Oral liquids' protein content not described.
Chen [6] 2015	Surgery for colorectal cancer.	**Water** was started on POD 1, water and a small amount of EN was taken repeatedly on POD 2. 1L of EN was taken repeatedly on POD 3 followed by a small amount of liquid food.	No early protein. Mortality not explicitly reported.
Chen [7] 2010	Surgery for colorectal cancer.	**Liquid diet** was began within 24 hours after surgery, and then gradually continued to solid diet.	Mortality not explicitly reported.
Da Fonseca [8] 2011	Colorectal resections.	**'Oral liquid diet**' commenced on POD 1.	Early protein *possible,* but not explicit in protocol. 'Oral liquid diet' protein content not described.

©2020 Gordon S. Doig, University of Sydney.

Dag [9] 2011	Colorectal surgery	Postoperative oral feeding commencing approximately 12 hours after the operation with a "fluid diet".	Early protein *possible,* but not explicit in protocol. 'Fluid diet' protein content not described.
Delaney [10] 2003	Patients scheduled for elective segmental intestinal or rectal resection by laparotomy.	On Postoperative Day (POD) 1, patients were encouraged to walk at least one circuit of the nursing floor (approximately 60 meters) up to five times, to sit out of bed between walks, and to do regular incentive spirometry. They were allowed **noncarbonated liquids *ad libitum*** and were offered **solid food** that evening if tolerating oral fluids.	Mortality not explicitly reported.
El Nakeeb [11] 2009	Elective colonic anastomosis.	Patients began **fluids** on the first postoperative day and advanced to a **regular diet** within the next 24–48 h, as tolerated (indicated by an absence of vomiting or abdominal distension).	Early protein *possible,* but not explicit in protocol. 'Fluids' protein content not described.
Feo [12] 2004	Colorectal resection.	Patients were '**allowed to drink**' the day after the operation, eat a **soft diet** the following day regardless of the passage of flatus, and were then advanced to solid food as tolerated.	Early protein *possible,* but not explicit in protocol. 'Fluids' protein content not described.
Han [13] 2012	Surgery for colorectal cancer.	**Liquid diet** was began within 6-8 hours.	Mortality not explicitly reported.
Han-Geurts [14] 2001	Elective abdominal surgery including open colonic surgery and transabdominal central vascular reconstruction procedures.	Patients were assigned to a patient-controlled (PC) diet after surgery of a fixed regimen (FR). Patients in the PC group chose when to start an oral diet. ***There was no motivation to start early.*** Patients in the FR group started water on POD 0 and 1. On POD 2 a 'liquid diet' was commenced. Liquid diet was defined as water, tea, coffee, lemonade.	No early protein.
Han-Geurts[15] 2007	Elective open colorectal or abdominal vascular surgery.	Patients were assigned to a conventional dietary regimen or a diet of their own choice. ***There was no motivation to start early.*** Patients in the conventional diet group started water on POD 0 and 1. POD 2 and 3, they continued with a 'liquid diet' defined as water, tea, coffee and lemonade.	No early protein.
Hartsell [16] 1997	Elective colorectal surgery.	Commence '**liquid diet**' on POD 1, advance to regular diet when they consumed 1L in 24 h.	Early protein *possible,* but not explicit in protocol. Protein content of 'liquid diet' not described.
He [17] 2016	Elective resection for colorectal carcinoma.	**Water** was began on POD 1, followed by 500ml of enteral nutrition on POD 2.	No early protein.<br>Mortality not explicitly reported.

©2020 Gordon S. Doig, University of Sydney.

Hoover [18] 2000	Extensive esophageal, gastroduodenal, biliary or pancreatic procedures.	Immediate post-op elemental diet via jejunostomy catheter vs. IV glucose.	Not lower GI tract surgery.<br>Mortality not explicitly reported.
Kemen [19] 1995	Upper GI malignancies.	On POD 1, patients were randomized to receive either the arginine, RNA, and omega-3 fatty acids supplemented diet or an isocaloric and isonitrogenous placebo diet.	Not lower GI tract surgery.<br>Both groups received early protein.
Lee [20] 2011	Laparoscopic colon surgery.	Patients were allowed to take water (less than 1 L) immediately after the operation. They progressed to ***semifluid diet*** on POD 1 and commenced a regular diet in 2 days after surgery.	Early protein *possible,* but not explicit in protocol. Protein content of 'semifluid diet' not described.
Li [21] 2006	Postoperative colorectal cancer	POD 1: **Began 5% Glucose 500ml through jejunostomy tube**; POD 2: Enteral Nutrition (Peptison, NUTRICIA) 1500ml to 2000ml and continued until POD 7.	No early protein.
Lidder [22] 2013	Colorectal surgery.	Pre-op carbohydrate and Post-op protein drink (factorial design). In the postoperative period patients were given polymeric nutritional supplement drink or placebo (600 ml/day) from the period immediately after their operation until discharge. Patients in the supplement group consumed Fortifresh (Numico, Zoetermeer, the Netherlands). All patients received **"free fluids permitted immediately after surgery *and* a light diet as tolerated by the patient."**	Both groups received early protein. This trial compares 'standard' early protein (light diet from POD 1) to 'standard' early protein (light diet from POD 1) *plus* protein supplement drink.
Lucha [23] 2005	Elective open GI surgery. Over half procedures were lower GI.	Early management consisted of bowel rest for 8 hours after completion of surgery followed by a **regular diet**. Traditional management consisted of bowel rest until the passage of flatus, followed by 24 hours of clear liquids and advancement to a regular diet.	Mortality not explicitly reported.
MacFie [24] 2000	Majority colorectal (94%)	Pre-op carbohydrate and Post-op protein drink (factorial design). On POD 1, patients were randomized to normal diet or normal diet plus protein supplement drink (Fortisip, Nutricia Ltd., Towbridge, Wiltshire, UK).	Both groups received early protein. This trial compares 'normal' early protein (normal diet from POD 1) to 'normal' early protein (normal diet from POD 1) *plus* protein supplement drink.

©2020 Gordon S. Doig, University of Sydney.

Nematihonar [25] 2018	Colorectal anastomosis.	Early feeding after surgery was initiated by **filtrate liquids** within 24 h after surgery. Over the next 24 h, the liquid diet was replaced by a normal diet in case tolerance was desirable.	Early protein *possible,* but not explicit in protocol. Protein content of 'filtrate liquids' not described. Normal diet introduced later than 24 h after surgery.
Nessim [26] 1999	Patients without stoma who underwent anorectal reconstructive surgery.	Study intervention (bowel confinement) patients received a clear liquid diet with loperamide 4 mg by mouth three times per day for three days, with codeine phosphate 30 mg by mouth four times per day for three postoperative days after the operation. Patients in the regular diet group began a regular diet on the day of surgery.	Mortality not explicitly reported.
Reissman [27] 1995	Elective colorectal surgery with bowel resection.	Patients in the early feeding group began a **clear liquid diet** on the first postoperative day and advanced to a regular diet within the next 24 to 48 hours, as tolerated (absence of vomiting or abdominal distention). Control group received NPO until resolution of ileus.	No early protein.
Pragatheeswarane [28] 2014	Elective open bowel surgery.	**Clear liquid** diet started at the 24 hour mark after surgery. Full fluid diet offered within 48 h and solid diet over next 24 h.	No early protein.
Qu [29] 2011	Surgery for colorectal cancer.	**Water** was began within 2 hours of surgery and then a small amount of **enteral nutrition** was began after 2 hours, with total amount less than 600ml.	Mortality not explicitly reported.
Ren [30] 2014	Surgery for colorectal cancer.	**Water** was began on POD 1, followed by water and 500ml of enteral nutrition on POD 2.	Mortality not explicitly reported.<br>No early protein.
Ryan [31] 1981	Elective colectomy.	Early postoperative jejunal feeding (n=9) of **elemental diet** (Vivonex HN, 42 g/L protein) compared to intravenous isotonic glucose (n=7).	Mortality not explicitly reported.
Sagar [32] 1979	Major GI surgery. (>70% lower GI tract surgery)	Starting POD 1, for the first 24 hours **elemental solution** (Flexical) was infused at half strength solution at 25 ml/hour. Thereafter, undiluted Flexical was infused at 25 ml/hour on the second postoperative day, 50 ml/hour on the third postoperative day, and 100 ml/hour on the fourth and fifth days. Conventional patients received NPO for two days.	Mortality not explicitly reported.
Sharma [33] 2013	Colorectal surgery.	Standard Hospital Diet includes **free fluids** allowed immediately postoperatively with a **standard diet** offered to all the patients from day 1.<br>Patients were randomized to Standard Hospital Diet or Standard Hospital Diet plus protein drink supplements (Pro-Cal).	Both groups received early protein. This trial compares 'normal' early protein (standard hospital diet from POD 1) to 'normal' early protein (standard hospital diet from POD 1) *plus* protein supplement drink.

©2020 Gordon S. Doig, University of Sydney.

Schroeder [34] 1991	Bowel resection. (>90% received lower GI tract procedures.)	On arrival back at the ward from the recovery room, the patient was assessed and immediate nasojejunal infusion with **full-strength Osmolite** (Ross Laboratories) at 50 mL/h via a continuous infusion pump was commenced. The control group received 5% dextrose plus oral fluids and food recommenced at the discretion of the clinical team, usually depended on the presence of bowel sounds and passage of flatus.	Mortality not explicitly reported.
Smedley [35] 2004	Lower GI tract surgery.	Pre-op carbohydrate and Post-op protein drink (factorial design). **Protocol does not prescribe early normal diet or early protein drink intake.** All patients received standard postoperative care with commencement of free fluids and reintroduction of normal diet without interference by the study team or protocol. Patients randomized to standard diet plus supplement were encouraged to drink Fortisip (Nutricia) ad libitum in small, frequent quantities between meals.	No early protein.
Soliani [36] 2001	Major surgery of the abdomen and pelvis.	Compares **early PN vs. early EN vs. early immunoenhanced EN**. No differences in time to receiving protein between groups (ePN, eEN and eEIN).	All three groups received early protein.
Wang [37] 2015	Postoperative colorectal cancer.	**Water** was began on POD 1, followed by water and 500ml of **enteral nutrition** on POD 2	Mortality not explicitly reported. No early protein.
Wang [38] 2013	Postoperative colorectal cancer.	POD 1: **Began small amount of water orally**; POD 2: progressed to Enteral Nutrition (Jevity, Abbott) 500 ml and water; POD 3: EN 1000 ml and Liquid diet on the third POD (Jevity, Abbott) 1000 ml , added Liquid diet on the fourth, and continued on the fifth POD .	No early protein.
Wu [39] 2007	Malnourished patients undergoing elective GI cancer surgery (65% had gastrectomy, 35% had colon or rectal surgery)	Randomized to three groups: Commenced on POD 1, compares 1) control **(5% and 10% glucose IV and later normal diet**) vs. 2) **early EN** vs. 3) **early PN**.	Mortality not explicitly reported for colorectal surgery patients.
Wu [40] 1996	Gastrointestinal disease surgery.	Randomized to three groups: Commenced on POD 1, compares 1) control (**5% and 10% glucose IV and later normal diet**) vs. 2) **early EN** vs. 3) **early PN**.	Mortality not explicitly reported. Abstract only.
Wu [41] 2000	Gastrointestinal disease surgery.	Randomized to three groups: Commenced on POD 1, compares 1) control (**5% and 10% glucose IV and later normal diet**) vs. 2) **early EN** vs. 3) **early PN**.	Mortality not explicitly reported. Abstract only.

©2020 Gordon S. Doig, University of Sydney.

Wu [42] 2012	Postoperative colorectal cancer	Started within 24 h of surgery: 500mls of **protein drink (Ensure, Abbott)**. POD 2: 1000 mls of protein drink; POD 3: 1500 mls of protein drink, continued until POD 7.<br>Compared to Pareneral Nutrition (glucose,amino acids and lipids) until POD 7. PN was commenced within 24 h of completion of surgery.	Both groups received early protein.
Xu [43] 2012	Postoperative colorectal cancer	**Water** was began within 6 hours after surgery, **enteral nutrition** was started on POD 1.	Mortality not explicitly reported.
Yang [44] 2013	Postoperative colorectal cancer	Oral intake of 30ml to 50ml **Ensure (US Abbott)** 6 to 12 hours post-surgery at 1- to 2-hours intervals. Progressed to 100ml to 200ml Ensure at 2- to 3- hours intervals on POD 2. POD 3 to 4; started with liquid diet and gradually changed to a regular diet .	Mortality not explicitly reported.
Zhou [45] 2006	Colorectostomy.	Early feeding patients were provided immediately **water** and gradually to a **liquid fiberless diet** after one day, and a **semi-liquid fiber diet** after three days. Control patients commenced nutrition after passage of flatus.	Mortality not explicitly reported.<br>Early protein *possible,* but not explicit in protocol. Protein content of 'liquid fiberless diet' not described.

CNKI: Identfied by searching China National Knowledge Infrastructure, **EN:** enteral nutrition, **PN:** refers to a parenteral nutrition solution containing protein.

©2020 Gordon S. Doig, University of Sydney.

eFigure 1. Risk of bias summary figure.

Study	Allocation concealment	Use of any form of blinding	Incomplete outcome data (loss to follow-up exceeds 10%)	Sequence generation*	Reporting bias**
Beier-Holgersen 1996	Unclear	Low	Unclear	Unclear	Unclear
Carr 1996	Low	Unclear	Unclear	Unclear	Unclear
Lau 2014	Low	Unclear	Low	Unclear	Unclear
Minig 2009	Low	Unclear	High	Unclear	Unclear
Mulrooney 2005	Unclear	Unclear	Unclear	Unclear	Unclear
Ortiz 1996	Unclear	Unclear	Unclear	Unclear	Unclear
Shen 2013	Unclear	Unclear	Unclear	Unclear	Unclear
Stewart 1998	Unclear	Unclear	Low	Unclear	Unclear

- ● Low risk of bias
- ● Unclear risk of bias
- ● High risk of bias

* Complete reporting of Sequence generation procedures helps improve confidence the study is not at high risk of selection bias and should be interpreted in context of Allocation concealment.
** Selective reporting is best interpreted in the context of an a priori published study Protocol. No studies reported publicly available a priori published Protocols.

©2020 Gordon S. Doig, University of Sydney.

eFigure 2. Funnel plot for publication bias. Primary outcome (mortality).

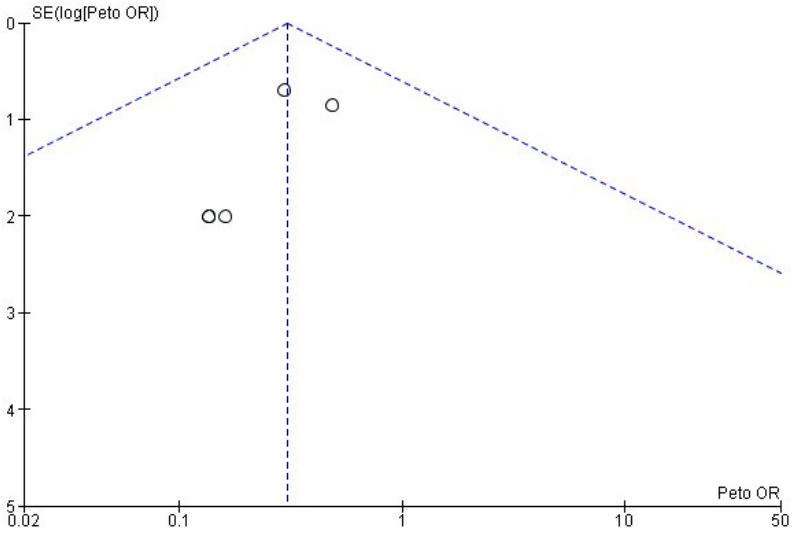

eFigure 3: Duration of hospital stay

Study or Subgroup	Early nutrition Mean [Days]	SD [Days]	Total	Control Mean [Days]	SD [Days]	Total	Weight	Mean Difference IV, Fixed, 95% CI [Days]
Carr 1996	9.8	6.6	14	9.3	2.8	14	2.8%	0.50 [-3.26, 4.26]
Lau 2014	5	2.6	50	7	4.9	54	17.6%	-2.00 [-3.49, -0.51]
Minig 2009	6.9	2.6	19	9.1	4.5	22	8.0%	-2.20 [-4.41, 0.01]
Mulrooney 2005	11.79	4.46	36	10.57	4.64	37	9.0%	1.22 [-0.87, 3.31]
Ortiz 1996	13.13	4.23	95	16.57	9.23	95	9.4%	-3.44 [-5.48, -1.40]
Shen 2013	16.15	2.53	41	19.69	2.31	41	35.7%	-3.54 [-4.59, -2.49]
Stewart 1998	9.36	4.11	40	10.08	2.55	40	17.5%	-0.72 [-2.22, 0.78]
**Total (95% CI)**			**295**			**303**	**100.0%**	**-2.12 [-2.74, -1.49]**

Heterogeneity: Chi² = 23.73, df = 6 (P = 0.0006); I² = 75%
Test for overall effect: Z = 6.63 (P < 0.00001)

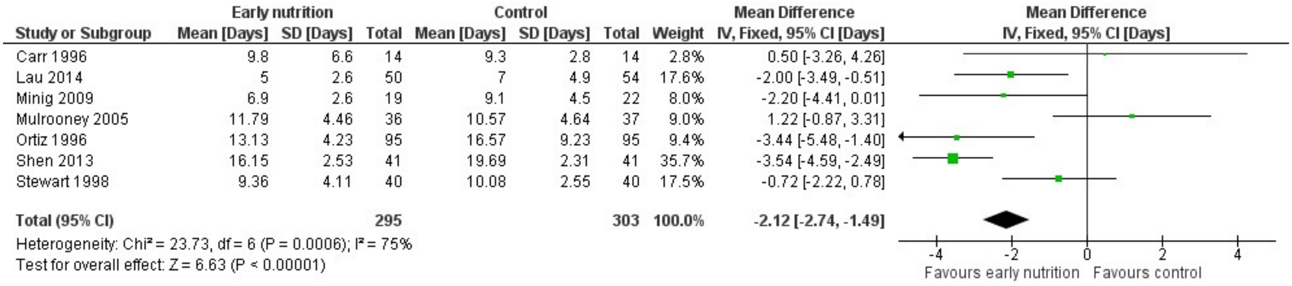

eFigure 4: Need for ICU admission

Study or Subgroup	Early nutrition Events	Total	Control Events	Total	Weight	Peto Odds Ratio Peto, Fixed, 95% CI
Beier-Holgersen 1996	2	30	6	30	39.5%	0.32 [0.07, 1.41]
Minig 2009	4	27	4	24	38.4%	0.87 [0.20, 3.90]
Ortiz 1996	2	95	2	95	22.1%	1.00 [0.14, 7.21]
**Total (95% CI)**		**152**		**149**	**100.0%**	**0.61 [0.24, 1.53]**
Total events	8		12			

Heterogeneity: Chi² = 1.18, df = 2 (P = 0.55); I² = 0%
Test for overall effect: Z = 1.06 (P = 0.29)

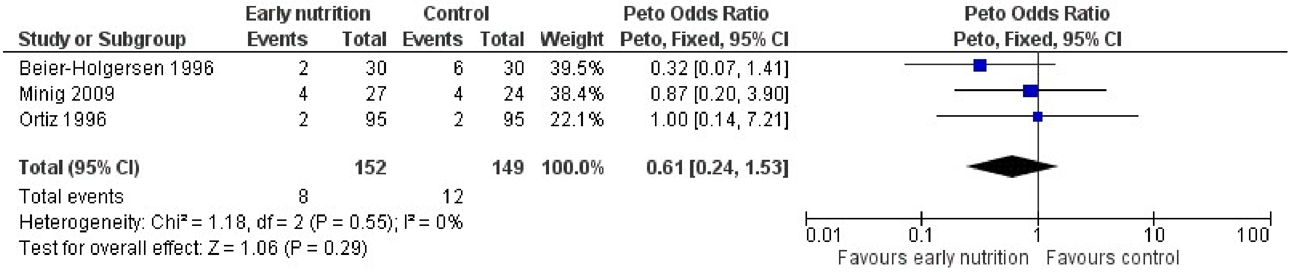

eFigure 5: Anastomotic leak/dehiscence

Study or Subgroup	Early nutrition Events	Total	Control Events	Total	Weight	Peto Odds Ratio Peto, Fixed, 95% CI
Beier-Holgersen 1996	2	30	4	30	30.4%	0.48 [0.09, 2.57]
Minig 2009	0	18	3	22	15.6%	0.15 [0.01, 1.52]
Mulrooney 2005	3	36	0	37	16.1%	8.05 [0.81, 79.93]
Ortiz 1996	2	93	4	93	32.3%	0.50 [0.10, 2.55]
Stewart 1998	1	40	0	40	5.5%	7.39 [0.15, 372.38]
**Total (95% CI)**		**217**		**222**	**100.0%**	**0.74 [0.30, 1.87]**
Total events	8		11			

Heterogeneity: Chi² = 7.78, df = 4 (P = 0.10); I² = 49%
Test for overall effect: Z = 0.63 (P = 0.53)

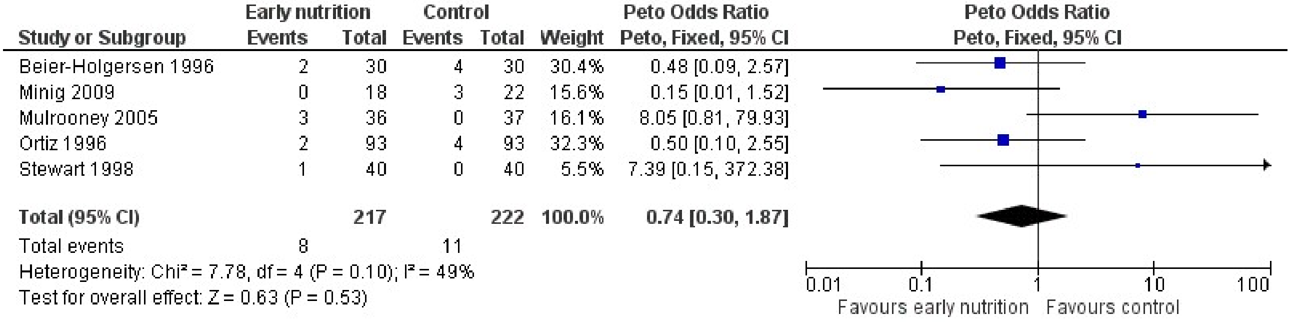

©2020 Gordon S. Doig, University of Sydney.

eFigure 6: Postoperative nausea and vomiting

Study or Subgroup	Early nutrition Events	Early nutrition Total	Control Events	Control Total	Weight	Peto Odds Ratio Peto, Fixed, 95% CI
Beier-Holgersen 1996	15	30	17	30	21.9%	0.77 [0.28, 2.10]
Carr 1996	1	14	7	14	8.6%	0.13 [0.03, 0.66]
Lau 2014	8	50	18	54	28.4%	0.40 [0.17, 0.97]
Minig 2009	10	18	12	22	14.5%	1.04 [0.30, 3.58]
Stewart 1998	14	40	14	40	26.6%	1.00 [0.40, 2.49]
**Total (95% CI)**		**152**		**160**	**100.0%**	**0.62 [0.38, 0.99]**
Total events	48		68			

Heterogeneity: Chi² = 6.39, df = 4 (P = 0.17); I² = 37%
Test for overall effect: Z = 2.02 (P = 0.04)

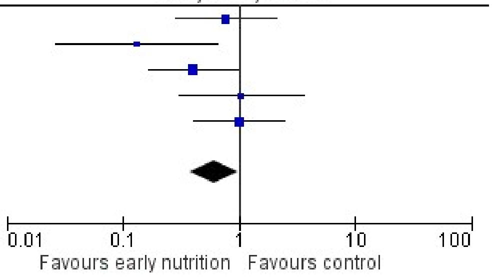

eFigure 7: Pneumonia

Study or Subgroup	Early nutrition Events	Early nutrition Total	Control Events	Control Total	Weight	Peto Odds Ratio Peto, Fixed, 95% CI
Beier-Holgersen 1996	1	30	2	30	12.7%	0.50 [0.05, 5.02]
Lau 2014	3	50	7	54	39.9%	0.45 [0.12, 1.66]
Mulrooney 2005	2	36	0	37	8.6%	7.82 [0.48, 127.47]
Ortiz 1996	2	93	2	93	17.2%	1.00 [0.14, 7.21]
Shen 2013	1	41	2	41	12.8%	0.50 [0.05, 4.99]
Stewart 1998	1	40	1	40	8.7%	1.00 [0.06, 16.27]
**Total (95% CI)**		**290**		**295**	**100.0%**	**0.73 [0.32, 1.66]**
Total events	10		14			

Heterogeneity: Chi² = 3.64, df = 5 (P = 0.60); I² = 0%
Test for overall effect: Z = 0.75 (P = 0.45)

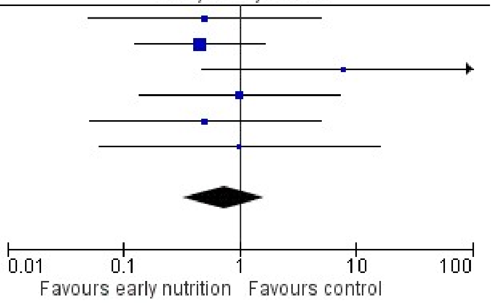

eFigure 8: Need for re-operation

Study or Subgroup	Early nutrition Events	Early nutrition Total	Control Events	Control Total	Weight	Peto Odds Ratio Peto, Fixed, 95% CI
Beier-Holgersen 1996	1	30	2	30	23.9%	0.50 [0.05, 5.02]
Lau 2014	2	50	3	54	39.5%	0.71 [0.12, 4.28]
Minig 2009	1	18	4	22	36.6%	0.32 [0.05, 2.08]
**Total (95% CI)**		**98**		**106**	**100.0%**	**0.49 [0.16, 1.51]**
Total events	4		9			

Heterogeneity: Chi² = 0.36, df = 2 (P = 0.84); I² = 0%
Test for overall effect: Z = 1.24 (P = 0.22)

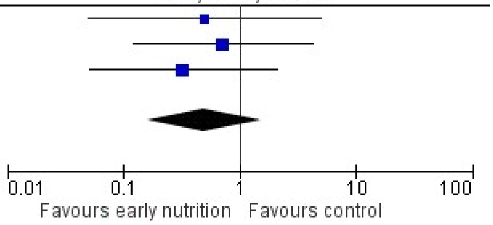

eFigure 9: Number of patients with intra-abdominal abscess/peritonitis

Study or Subgroup	Early nutrition Events	Early nutrition Total	Control Events	Control Total	Weight	Peto Odds Ratio Peto, Fixed, 95% CI
Beier-Holgersen 1996	0	30	2	30	18.4%	0.13 [0.01, 2.14]
Lau 2014	1	50	4	54	44.9%	0.31 [0.05, 1.86]
Minig 2009	0	18	2	22	18.0%	0.15 [0.01, 2.60]
Mulrooney 2005	0	36	0	37		Not estimable
Ortiz 1996	0	93	1	93	9.4%	0.14 [0.00, 6.82]
Shen 2013	0	41	1	41	9.4%	0.14 [0.00, 6.82]
**Total (95% CI)**		**268**		**277**	**100.0%**	**0.20 [0.06, 0.66]**
Total events	1		10			

Heterogeneity: Chi² = 0.43, df = 4 (P = 0.98); I² = 0%
Test for overall effect: Z = 2.63 (P = 0.008)

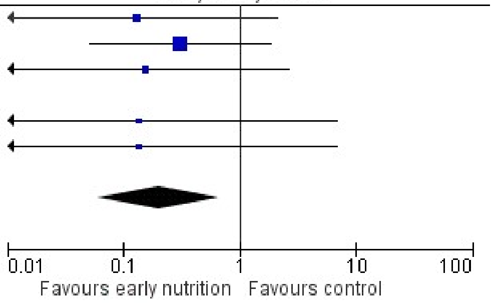

©2020 Gordon S. Doig, University of Sydney.

eFigure 10: Number of patients with serious post-operative complications

	Early nutrition		Control			Odds Ratio
Study or Subgroup	Events	Total	Events	Total	Weight	M-H, Fixed, 95% CI
Beier-Holgersen 1996	6	30	15	30	18.8%	0.25 [0.08, 0.79]
Lau 2014	14	50	24	54	26.0%	0.49 [0.21, 1.10]
Minig 2009	8	18	11	22	8.6%	0.80 [0.23, 2.79]
Ortiz 1996	17	93	18	93	23.0%	0.93 [0.45, 1.94]
Shen 2013	2	41	8	41	11.9%	0.21 [0.04, 1.07]
Stewart 1998	10	40	10	40	11.7%	1.00 [0.36, 2.75]
**Total (95% CI)**		**272**		**280**	**100.0%**	**0.60 [0.40, 0.89]**
Total events	57		86			

Heterogeneity: Chi² = 6.65, df = 5 (P = 0.25); I² = 25%
Test for overall effect: Z = 2.54 (P = 0.01)

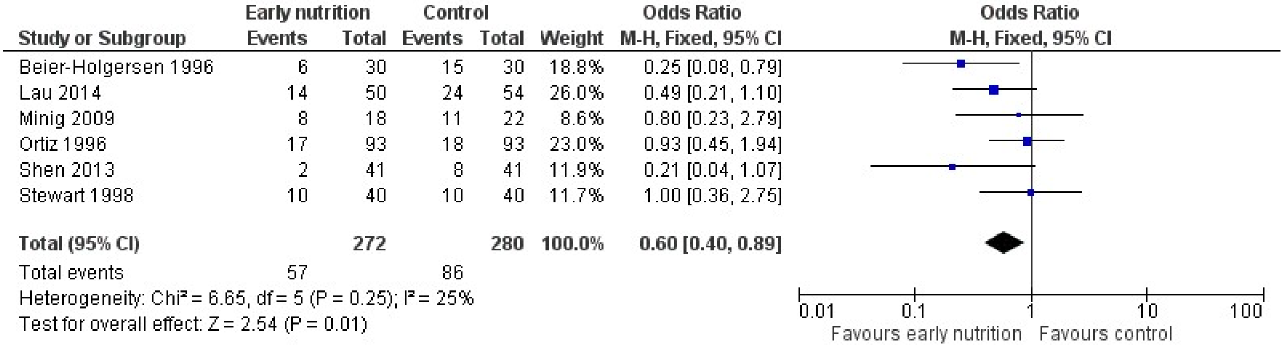

eFigure 11: Number of patients with a post-operative infection

	Early nutrition		Control			Peto Odds Ratio
Study or Subgroup	Events	Total	Events	Total	Weight	Peto, Fixed, 95% CI
Beier-Holgersen 1996	2	30	14	30	46.5%	0.13 [0.04, 0.42]
Carr 1996	0	14	3	14	10.8%	0.12 [0.01, 1.21]
Minig 2009	0	18	3	22	11.0%	0.15 [0.01, 1.52]
Shen 2013	2	41	7	41	31.6%	0.29 [0.07, 1.15]
**Total (95% CI)**		**103**		**107**	**100.0%**	**0.17 [0.08, 0.37]**
Total events	4		27			

Heterogeneity: Chi² = 0.88, df = 3 (P = 0.83); I² = 0%
Test for overall effect: Z = 4.48 (P < 0.00001)

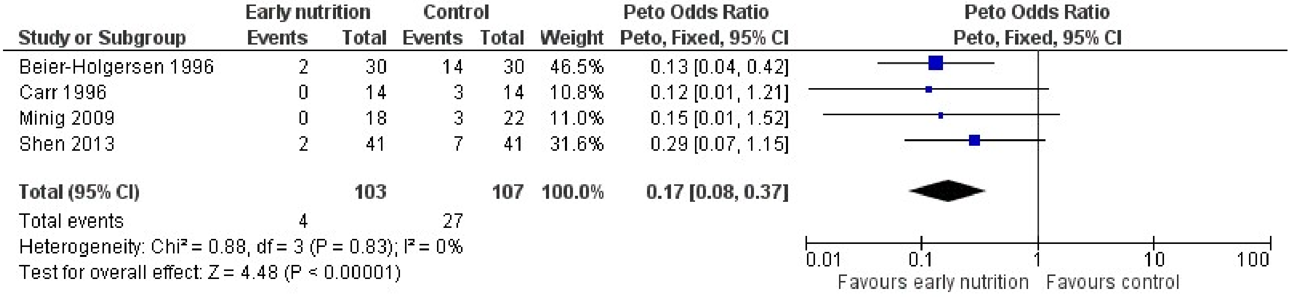

©2020 Gordon S. Doig, University of Sydney.

eFigure 12: Stratified analysis of duration of hospital stay

	Early nutrition			Control				Mean Difference
Study or Subgroup	Mean [Days]	SD [Days]	Total	Mean [Days]	SD [Days]	Total	Weight	IV, Fixed, 95% CI [Days]
**5.2.1 Enteral feeding tube**								
Carr 1996	9.8	6.6	14	9.3	2.8	14	2.8%	0.50 [-3.26, 4.26]
Mulrooney 2005	11.79	4.46	36	10.57	4.64	37	9.0%	1.22 [-0.87, 3.31]
**Subtotal (95% CI)**			**50**			**51**	**11.8%**	**1.05 [-0.77, 2.87]**
Heterogeneity: Chi² = 0.11, df = 1 (P = 0.74); I² = 0%								
Test for overall effect: Z = 1.13 (P = 0.26)								
**5.2.2 Solid diet**								
Lau 2014	5	2.6	50	7	4.9	54	17.6%	-2.00 [-3.49, -0.51]
Minig 2009	6.9	2.6	19	9.1	4.5	22	8.0%	-2.20 [-4.41, 0.01]
Ortiz 1996	13.13	4.23	95	16.57	9.23	95	9.4%	-3.44 [-5.48, -1.40]
Stewart 1998	9.36	4.11	40	10.08	2.55	40	17.5%	-0.72 [-2.22, 0.78]
**Subtotal (95% CI)**			**204**			**211**	**52.5%**	**-1.86 [-2.73, -1.00]**
Heterogeneity: Chi² = 4.65, df = 3 (P = 0.20); I² = 35%								
Test for overall effect: Z = 4.22 (P < 0.0001)								
**5.2.3 Protein drink supplement**								
Shen 2013	16.15	2.53	41	19.69	2.31	41	35.7%	-3.54 [-4.59, -2.49]
**Subtotal (95% CI)**			**41**			**41**	**35.7%**	**-3.54 [-4.59, -2.49]**
Heterogeneity: Not applicable								
Test for overall effect: Z = 6.62 (P < 0.00001)								
**Total (95% CI)**			**295**			**303**	**100.0%**	**-2.12 [-2.74, -1.49]**
Heterogeneity: Chi² = 23.73, df = 6 (P = 0.0006); I² = 75%								
Test for overall effect: Z = 6.63 (P < 0.00001)								
Test for subgroup differences: Chi² = 18.98, df = 2 (P < 0.0001), I² = 89.5%								

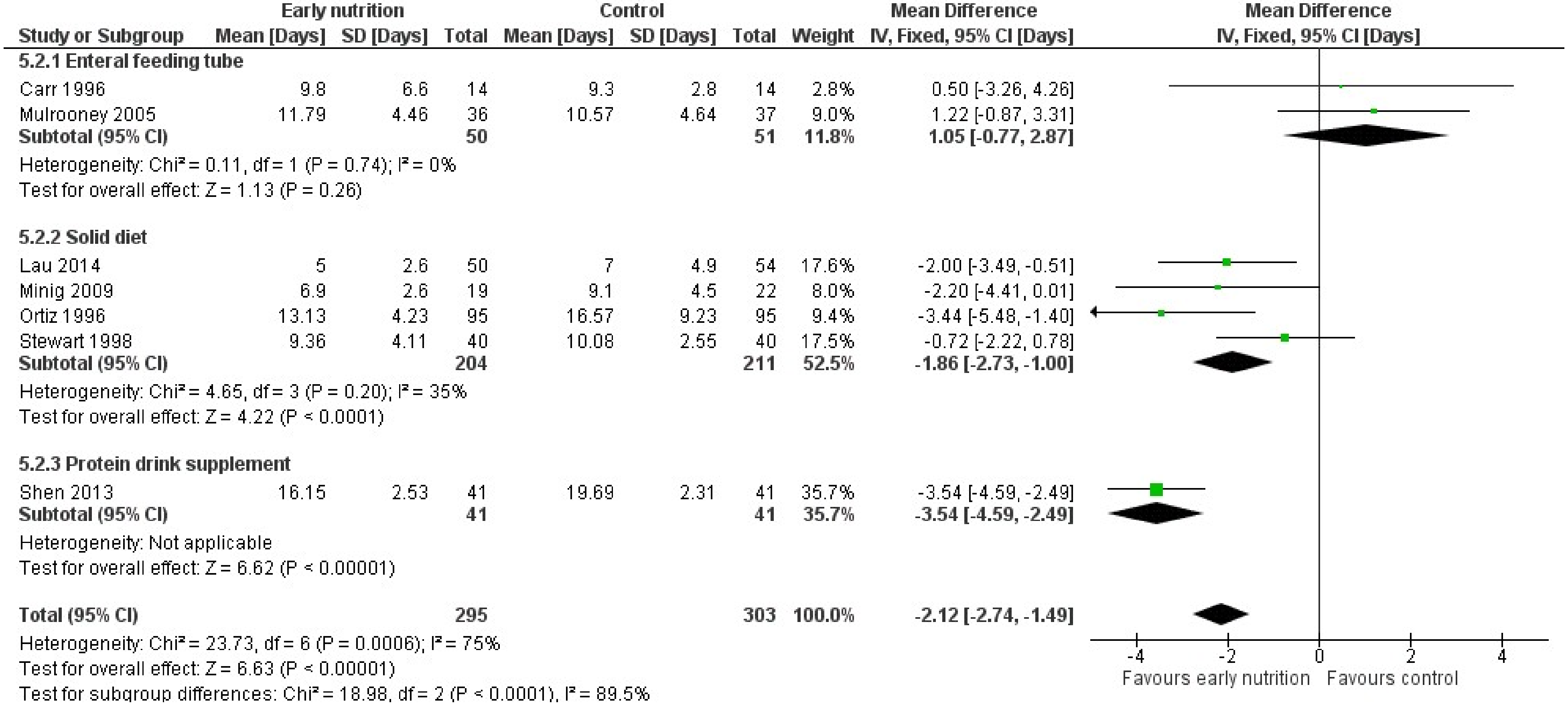

©2020 Gordon S. Doig, University of Sydney.

Reference List

1. Boelens PG, Houdijk APJ, Fonk JCM et al. Glutamine-enriched enteral nutrition increases HLA-DR expression on monocytes of trauma patients. Journal of Nutrition 2002;132(9):2580-2586.

2. Behrns KE, Kircher AP, Galanko JA, Brownstein MR, Koruda MJ. Prospective randomized trial of early initiation and hospital discharge on a liquid diet following elective intestinal surgery. J Gastrointest Surg 2000;4(2):217-221.

3. Binderow SR, Cohen SM, Wexner SD, Nogueras JJ. Must early postoperative oral intake be limited to laparoscopy? Dis Colon Rectum 1994;37(6):584-589.

4. Cao A. Application of early intestinal nutrition after colon and rectal cancer. Today Nurse 2009;9:42-43.

5. Chatterjee S, Bala SK, Chakraborty P et al. A comparative study between early enteral feeding (within 24 hours) versus conventional enteral feeding after enteric anastomosis. Bangladesh Journal of Medical Science 2012;11(4):273-283.

6. Chen J, Wuo W. Effect of early oral enteral nutrition on postoperative immune function and quality of life in patients with colorectal cancer. Chin J General Surg 2015;24(12):1774-1777.

7. Chen M, Cui H, Chen Y, Jai G. Clinical study on early postoperative feeding of colorectal cancer. J Pract Oncology 2010;25(4):453-454.

8. da Fonseca LM, Profeta da Luz MM, Lacerda-Filho A, Correia MI, Gomes da SR. A simplified rehabilitation program for patients undergoing elective colonic surgery--randomized controlled clinical trial. Int J Colorectal Dis 2011;26(5):609-616.

9. Dag A, Colak T, Turkmenoglu O, Gundogdu R, Aydin S. A randomized controlled trial evaluating early versus traditional oral feeding after colorectal surgery. Clinics (Sao Paulo) 2011;66(12):2001-2005.

10. Delaney CP, Zutshi M, Senagore AJ, Remzi FH, Hammel J, Fazio VW. Prospective, randomized, controlled trial between a pathway of controlled rehabilitation with early ambulation and diet and traditional postoperative care after laparotomy and intestinal resection. Dis Colon Rectum 2003;46(7):851-859.

11. El NA, Fikry A, El MT et al. Early oral feeding in patients undergoing elective colonic anastomosis. Int J Surg 2009;7(3):206-209.

12. Feo CV, Romanini B, Sortini D et al. Early oral feeding after colorectal resection: a randomized controlled study. ANZ J Surg 2004;74(5):298-301.

13. Han L. Patients with colorectal cancer do not have a nasogastric tube before surgery and eat early: Clinical study. Journal of Tianjin Medical University 2012;18(3):118-120.

14. Han-Geurts IJ, Jeekel J, Tilanus HW, Brouwer KJ. Randomized clinical trial of patient-controlled versus fixed regimen feeding after elective abdominal surgery. Br J Surg 2001;88(12):1578-1582.

15. Han-Geurts IJ, Hop WC, Kok NF, Lim A, Brouwer KJ, Jeekel J. Randomized clinical trial of the impact of early enteral feeding on postoperative ileus and recovery. Br J Surg 2007;94(5):555-561.

16. Hartsell PA, Frazee RC, Harrison JB, Smith RW. Early postoperative feeding after elective colorectal surgery. Arch Surg 1997;132(5):518-520.

©2020 Gordon S. Doig, University of Sydney.

17. He Q. Effect of early postoperative oral enteral nutrtion on postoperative immunity and intenstinal mucosal barrier in patients with colorectal cancer. Chin J Trad West Med Dig 2016;24(4):292-294.

18. Hoover HC, Jr., Ryan JA, Anderson EJ, Fischer JE. Nutritional benefits of immediate postoperative jejunal feeding of an elemental diet. Am J Surg 1980;139(1):153-159.

19. Kemen M, Senkal M, Homann HH et al. Early postoperative enteral nutrition with arginine-omega-3 fatty acids and ribonucleic acid-supplemented diet versus placebo in cancer patients: an immunologic evaluation of Impact. Crit Care Med 1995;23(4):652-659.

20. Lee TG, Kang SB, Kim DW, Hong S, Heo SC, Park KJ. Comparison of early mobilization and diet rehabilitation program with conventional care after laparoscopic colon surgery: a prospective randomized controlled trial. Dis Colon Rectum 2011;54(1):21-28.

21. Li Y, Wu X, Luo W, Zhang M, Huang X, Wu L. The study of clinical application of early postoperative enteral nutrition after colorectalectomy. West China Medical Journal 2006;21(1):26-27.

22. Lidder P, Thomas S, Fleming S, Hosie K, Shaw S, Lewis S. A randomized placebo controlled trial of preoperative carbohydrate drinks and early postoperative nutritional supplement drinks in colorectal surgery. Colorectal Dis 2013;15(6):737-745.

23. Lucha PA, Jr., Butler R, Plichta J, Francis M. The economic impact of early enteral feeding in gastrointestinal surgery: a prospective survey of 51 consecutive patients. Am Surg 2005;71(3):187-190.

24. MacFie J, Woodcock NP, Palmer MD, Walker A, Townsend S, Mitchell CJ. Oral dietary supplements in pre- and posoperative surgical patients: A prospective and randomized clinical trial. Nutrition 2019;16:723-728.

25. Nematihonar B, Salimi S, Noorian V, Samsami M. Early Versus Delayed (Traditional) Postoperative Oral Feeding in Patients Undergoing Colorectal Anastomosis. Adv Biomed Res 2018;7:30.:30.

26. Nessim A, Wexner SD, Agachan F et al. Is bowel confinement necessary after anorectal reconstructive surgery? A prospective, randomized, surgeon-blinded trial. Dis Colon Rectum 1999;42(1):16-23.

27. Reissman P, Teoh TA, Cohen SM, Weiss EG, Nogueras JJ, Wexner SD. Is early oral feeding safe after elective colorectal surgery? A prospective randomized trial. Ann Surg 1995;222(1):73-77.

28. Pragatheeswarane M, Muthukumarassamy R, Kadambari D, Kate V. Early oral feeding vs. traditional feeding in patients undergoing elective open bowel surgery-a randomized controlled trial. J Gastrointest Surg 2014;18(5):1017-1023.

29. Qu Q, Shi W, Li H. Effect of early oral feeding on postoperative recovery of patients with colorectal cancer. Chinese Journal of Gerontology 2012;2041-2043.

30. Ren H. Application value of early postoperative oral enteral nutrition in patients with colorectal cancer. The Practical Journal of Cancer 2014;29(10):1226-1228.

31. Ryan JA, Jr., Page CP, Babcock L. Early postoperative jejunal feeding of elemental diet in gastrointestinal surgery. Am Surg 1981;47(9):393-403.

32. Sagar S, Harland P, Shields R. Early postoperative feeding with elemental diet. Br Med J 1979;1(6159):293-295.

©2020 Gordon S. Doig, University of Sydney.

33. Sharma M, Wahed S, O'Dair G, Gemmell L, Hainsworth P, Horgan AF. A randomized controlled trial comparing a standard postoperative diet with low-volume high-calorie oral supplements following colorectal surgery. Colorectal Dis 2013;15(7):885-891.

34. Schroeder D, Gillanders L, Mahr K, Hill GL. Effects of immediate postoperative enteral nutrition on body composition, muscle function, and wound healing. Journal of Parenteral & Enteral Nutrition 1991;15(4):376-383.

35. Smedley F, Bowling T, James M et al. Randomized clinical trial of the effects of preoperative and postoperative oral nutritional supplements on clinical course and cost of care. Br J Surg 2004;91(8):983-990.

36. Soliani P, Dell'Abate P, Del RP et al. [Early enteral nutrition in patients treated with major surgery of the abdomen and the pelvis]. Chir Ital 2001;53(5):619-632.

37. Wang D, Zhong B, Zhao P, Liu X, Zhou Y. Effects of early enteral feeding on clinical outcomes and immune funciton in patients after colorectal cancer surgery. Chin J General Surg 2015;30(1):38-41.

38. Wang Z, Shong B, Xiang J, Zhou Y, Wang D. Effect of early oral enteral nutrition on clinical outcomes after colorectal cancer surgery. Chin J Gastrointest Surg 2013;16(8):735-738.

39. Wu GH, Zhang YW, Pan HT, Zhang B, Liu ZH, Wu ZH. A randomized controlled trial of posoperative artificial nutrition in malnourished patients with gastrointenstinal chancer. Chin J Gastrointest Surg 2007;10(6):546-549.

40. Wu GH, Jin DY, Wu ZH, Huang DX, Wu ZG. Study on the efficacy and safety of intestinal nutrition in the early stage after gastrointestinal surgery. Parenteral and Enteral Nutrition (China) 1996;3(2):86-87.

41. Wu GH, Zhang YW, Wu ZH. Study on the nutritional efficacy of the intestines in the early stage after the surgery of the digestive tract tumor. China Journal of Clinical Nutrition 2000;8(1):56.

42. Wu K, Ma J, Wang L. Clinical utility of postoperative early enteral nutrition after laparoscopic radical resection for colorectal cancer. J Dig Oncol 2012;4(3):180-182.

43. Xu L, Wan Z, Guo J et al. Influence of early oral feeding on serum albumin and inflammatory cytokines after rectal cancer surgery. J Clin Surg 2012;20(4):244-246.

44. Yang D, He W, Wang L et al. Effect of postoperative early enteral nutrition on the recovery of humoral immune function in patients with colorectal carcinoma undergoing elective resection. Chin J Gastrointest Surg 2013;6(11):1051-1054.

45. Zhou T, Wu XT, Zhou YJ, Huang X, Fan W, Li YC. Early removing gastrointestinal decompression and early oral feeding improve patients' rehabilitation after colorectostomy. World J Gastroenterol 2006;12(15):2459-2463.

< ------ last page------- >

©2020 Gordon S. Doig, University of Sydney.
